# 

**Published:** 1850-07

**Authors:** 


### NOTICE TO READERS AND CORRESPONDENTS.

Original communications have been received from Drs. P. A. Allaire, Thos. I, Sheldon, Thos.D. Washburn, C. C. Warner, A. Garwood, W. Brooks, I. S. Du Kate Jno. A, Haldeman and C. D. Chambers, most of which will be published in our next, with other articles already noticed.

We have received from Messrs. Lea & Blanchard, through S. C. Griggs & Co., the 2d number of Maclise's Surgical Anatomy, and a new edition of Churchill's Treatise on the Diseases of Females.

Also, from Barrington & Haswell, through J. Keene, Jr., & Bro., Ricord, on Venereal Diseases, & Bell, on Baths and the Watery Regimen.

A number of specimens of "Extra Medicines" have been received from Philip Scheiflin & Co., many of which are of superior quality to any we have before seen.

For want of room, we are unable to acknowledge the receipt of a number of pamphlets, catalogues and journals that shall be noticed and acknowledged in our next number.

The proceedings of Wisconsin State Medical Society, and of the N. W. Society of Wisconsin, have been received.

---

### VACCINE VIRUS

Can be obtained by sending One Dollar, post-paid, to the address of the Editors of this Journal. The Virus will be enclosed in a letter, and sent by the return mail

### LIST OF RECEIPTS FOR MAY AND JUNE, 1850.

### INDIANA.

| | |
|---|---|
| Drs. R. K. Long, | $4 00 |
| S. Loftin, | 2 00 |
| T. Bullard, | 2 00 |
| R. Jackson, | 2 00 |
| Dr. Bond, | 6 00 |
| S. M. Linton, | 4 00 |
| C. V. Jones, | 4 00 |
| W. C. Thompson, | 2 00 |
| W. J. Walker, | 2 00 |
| A. Bigelow, | 4 00 |
| E. Johnson, | 2 00 |
| Jno. H. Wiley, | 2 0 |
| H. W. Mann, | 2 00 |
| John Hunt, | 2 00 |
| Jno. M. Clarke, | 4 00 |
| B. T. Cox, | 3 00 |
| Dr. Perkins, | 2 00 |
| G. M. Walker, | 2 00 |
| C. Brown (ballance), | 50 |
| S. Anthony, | 2 0 |
| R. Winton, | 2 00 |
| U. Farquhar, | 2 00 |
| D. McKyssick, | 2 00 |
| R. C. Moore, | 2 00 |
| J. De Pew, | 2 00 |
| H. Goodwin, | 4 00 |
| T. T. Butler, | 4 00 |
| Israel Haines, | 2 00 |
| G. Perry, | 2 00 |
| J. A. Martin, | 2 00 |
| Wm. Carpenter, | 2 0 |
| Thos. Chesnut, | 6 00 |
| D. H. Crouse, | 2 0 |
| H. T. Snook, | 2 00 |
| S. Reifenberick, | 2 00 |
| W. H. Prestley, | 4 00 |
| H. E. Ellis, | 2 00 |
| J. S. Colling, | 3 00 |
| D. J. Shirley, | 2 00 |
| D. A. Gall, | 4 00 |
| L. W. Hess, | 2 00 |
| R. M. Gilkeson, | 2 00 |
| Seneca Ball, | 2 00 |
| A. M. Porter, | 2 00 |
| Jas. Ritchey, | 2 00 |
| Samuel Kelley, | 4 00 |
| J. F. Fraley, | 5 00 |
| T. S. Davidson, | 2 00 |
| A. M. C. Hawes, | 6 00 |
| H. F. Cannon, | 6 00 |
| S. T. Clarke, | 4 00 |

### ILLINOIS.

| | |
|---|---|
| Drs. W. Brooks, | 2 75 |
| J. Gregory, | 2 00 |
| Jas. S. Whitmire, | 2 00 |
| E. E. Webster, | 2 00 |
| A. Smith, | 2 00 |
| R. Morris, | 2 00 |
| J. F. Daggett, | 4 00 |
| H. Cutler, | 2 00 |
| H. Russell, | 2 00 |
| S. G. Baldwin, | 5 00 |
| W. Henry Davis, | 2 00 |
| Wood & Wilson, | 2 00 |
| Samuel G. Mans, | 2 00 |
| T. L. Fitch, | 2 00 |
| J. S. Mans, | 3 00 |
| W. C. Quigly. | 2 00 |
| J. P. Walker, | 1 00 |
| D. Wheeler, | 2 00 |
| O. George, | 2 00 |
| U. P. Golliday, | 2 00 |
| C. Skillman, | 4 00 |
| W. W. Southwick, | 2 00 |

### WISCONSIN.

| | |
|---|---|
| Drs. Clarke and Rice, | 2 00 |
| J. T. Buckley, | 3 00 |
| G. D. Maxson, | 2 00 |
| P. P. Greene, | 2 00 |
| M. M. Barber, | 4 00 |
| Benj. Morrison, | 4 00 |
| J. D. Hawes, | 4 00 |
| T. G. Cole, | 2 00 |
| J. H. Turner, | 2 00 |
| A. & E. N. Clarke, | 2 00 |
| W. Gorham, | 2 00 |

### OHIO.

| | |
|---|---|
| Drs. H. Burritt, | 2 00 |
| S. B. Olney, | 2 00 |

### IOWA.

| | |
|---|---|
| Drs. J. D. Blanchard, | 3 00 |
| Asa Horr, | 6 00 |
| F. Brady, | 2 00 |

### MICHIGAN.

| | |
|---|---|
| Dr. U. Upjohn, | 3 00 |

### ARKANSAS.

| | |
|---|---|
| W. Wilson, Esq., | 4 00 |
| Dr. Williams, | 2 00 |

